# Disruption to vegetable food systems during the COVID‐19 pandemic in the Lao People's Democratic Republic

**DOI:** 10.1111/sjtg.12447

**Published:** 2022-08-01

**Authors:** Chanthaly Syfongxay, Daovy Kongmanila, Phonevilay Sinavong, Silinthone Sacklokham, Kim Suzanne Alexander

**Affiliations:** ^1^ Provincial Department of Agriculture and Forestry Xiengkhoung Province Lao PDR; ^2^ Faculty of Agriculture National University of Laos Vientiane Lao PDR; ^3^ National Agriculture and Forestry Research Institute Ministry of Agriculture and Forestry Vientiane Lao PDR; ^4^ Ministry of Education and Sports Vientiane Lao PDR; ^5^ University of New England Armidale New South Wales Australia

**Keywords:** COVID‐19, vegetable supplies, Lao PDR, food supply, food systems

## Abstract

Globally, the COVID‐19 (SARSCoV‐2) pandemic has affected human health and the flow of goods and services in many sectors, with significant social and economic consequences and repercussions. COVID‐19 lockdowns have disrupted food systems; impacting farmers, food producers, traders and consumers. Using a food system approach, disruptions to and the resilience of vegetable food production and trade was analysed. Representatives of traditional farming systems in Lao PDR producing and trading vegetables were involved. Over 350 farmers, 60 wholesalers, 50 retailers and 70 consumers were surveyed to determine the disruptions to vegetable supplies in terms of quantities traded, prices and income fluctuations. Findings revealed significant impacts on trading capacity and consequent reductions in incomes, prices, purchases, transport and sales of produce. However, livelihoods resumed as soon as the lockdown lifted. Traditionally, vegetable production and trading are a woman's tasks and hence women were the most affected by the disruptions. With trading contractions, the stability of the food supply was threatened, but only temporarily, indicating that a traditional, resilient farming system based on lower population densities, lower input requirements and lower productivity could adapt to novel disruptions in the short term.

## Introduction

Since 2019, the COVID‐19 (SARSCoV‐2) viral pandemic has spread rapidly across the world with serious health, social and economic consequences and repercussions; impacting lives and livelihoods particularly at the household level (Market Development Facility, 2021; OECD, 2020a; Sidaway, 2021). As the pandemic engulfed the world, international borders abruptly closed and social distancing requirements and restrictions to movement were imposed in some form or other within and between countries (Laborde *et al*., 2020). These restrictions negatively impacted the world economy and the flow of goods and services in many sectors, including global food and agriculture sectors. Disruptions in the supply of agri‐food products to markets and consumers both within and across countries occurred (OECD, 2020b). In many countries, disruptions affected food production and demand, and adversely affected food availability (OECD, 2020a). Health, business and agricultural supply chains continue to be significantly and adversely affected by ongoing measures to curtail the spread of arising COVID‐19 variants (CIDRAP, 2022).

Food systems and access to food depends on functioning supply chains involving the movement of goods through production systems, storage and distribution, processing and packaging, and retail and markets (Cable *et al*., 2021). Food systems feature complex interactions between farmers and food producers, local and federal governments, and consumers. Economic, environmental and societal factors are known to impact food availability, access, affordability and food safety (Cable *et al*., 2021; Committee on World Food Security, 2017). COVID‐19 disruptions have impacted food production and food security, reducing access for many people to sufficient, safe, nutritious food (Cable, 2021; Sumner *et al*., 2020). Food system disruptions further impact the United Nations Sustainable Development Goals (SDGs).

FAO (2020) conducted a rapid review of COVID‐19 impacts on food systems in the Asia Pacific region. Considerable impacts were identified for food security and nutrition, and on food supply chains and regional trade. Many elements of food and livestock production have been affected, so too fisheries, natural resource management, food safety, animal‐to‐human health risks, and movements of transboundary pests and diseases (FAO, 2020).

Dixon *et al*. (2021) investigated the effects of the COVID‐19 pandemic on regional agri‐food systems in rural Asia and found that rural livelihoods and food security were impacted by disruptions to local labour markets, creating difficulties in ensuring perishable farm produce was transported to markets, and found disruptions to input supply chains such as seeds and fertilizers. According to ADB (2020), in Asia and the Pacific regions, lockdowns have impacted all stages of food supply chains, including food supply and demand, food prices, marketing, logistics and trading systems. Concurrently, measures to contain COVID‐19 in the Asia Pacific region have decreased labour mobility, including local and immigrant farm labourers and factory process workers, and decreased availability of inputs such as seeds and fertilizers, which has affected the availability of food and increased the cost of production and transport, placing upward pressure on food prices (ADB, 2020; Suhardiman *et al*., 2021). Rising food prices have also resulted from increased shipping costs, adverse weather conditions in exporting nations and increasing demand as governments have stockpiled commodities (Market Development Facility, 2021). Panic buying and hoarding have driven up prices of certain food staples. National trade restrictions and food policies have also contributed to fluctuations in food prices. Restrictions in movement of traders, middlepersons and farmers have impacted sales of farmer produce, particularly cash crops and horticulture as well as reducing the availability of certain food products (World Food Programme, 2020). Continued uncertainty about supply chain disruptions and economic recovery to pre‐COVID‐19 levels in the Asia Pacific region has resulted in pessimism among lenders and financial markets (Sanderson *et al*., 2020). This suggests that short term lockdowns can have longer term implications for food systems.

In the Asia Pacific, livelihoods have been disrupted as unemployment has increased in many rural areas, largely affecting daily labourers (World Food Programme, 2020). Migrant workers have been forced to return to rural areas, further impacting livelihoods through reduced remittances, thus significantly affecting off‐farm income dependant households (IWMI & NAFRI, 2021; World Food Programme, 2020). Dixon *et al*. (2021) found that female farmers in the Asia Pacific were most affected by COVID‐19 because of changes to farm work, fewer off‐farm income opportunities, reduced livelihoods and entrepreneurial activities, as well as food and economic insecurity.

Household food consumption has changed in response to the pandemic where incomes declined and access and mobility to retail food outlets and other sources of food has been curtailed, particularly when restrictive stay at home measures have been imposed (ADB, 2020; World Food Programme, 2020). The sale of perishable crops has been more affected, due to lower consumer demand, income losses and closure of food service industries (ADB, 2020). As countries continue to use lockdown strategies to manage the COVID‐19 pandemic to protect their populations' health, food supply systems continue to be at risk with implications for food security, particularly for poor rural people.

The aim of our article is to investigate the impacts of COVID‐19 lockdowns on food system activities, productivity, trade and consumption, and livelihood outcomes, using a food system approach. The disruption to and the resilience of vegetable food production, consumption and trade was analysed. Notably, vegetable supply chains in Xiengkhoung Province, the Lao People's Democratic Republic (Lao PDR) provide important nutrients as dietary components of rice‐based diets. We identified measures taken by smallholder farmers, traders, retail agents and consumers to ensure a continuous supply of vegetables to maintain food security during the first COVID‐19 lockdown period in 2020. We interrogated participants' responses about food system activities undertaken to mitigate the impacts of lockdowns and restricted movements of people and produce during lockdown. We questioned what decisions and activities were undertaken by key actors in the vegetable food system to maintain food security and livelihoods and to build a more resilient system for future events. By understanding changes to vegetable food system activities, we endeavour to supplement knowledge on food system outcomes and the status of food security during and after the first COVID‐19 lockdown in Lao PDR.

The article is structured as follows. We begin by introducing the food systems framework as the theoretical approach in understanding the systemic shocks to food supplies brought by COVID‐19 lockdowns and the changes to production and consumption practices. We follow that by outlining the research design, study sites and research methods used to explore the complexities faced by participants in vegetable supply chains due to COVID‐19 lockdown disruptions. We provide an account of how the COVID‐19 pandemic impacted the vegetable food systems in Xiengkhoung Province, during the period March–May 2020. We conclude with policy recommendations that contribute towards ensuring future food security and minimizing immediate negative impacts that may be brought about by future pandemics. This article adds to extant literature on the short‐term impacts of COVID‐19 on livelihoods of smallholder farmers relying on marginal returns to labour in Southeast Asia. It also illustrates altered behaviours, in response to livelihood threats, by rural people vulnerable to food insecurity living on minimal incomes in Lao PDR.

## Conceptualizing food systems

A food system can be considered as the chain of activities from production to consumption, the outcomes of these activities, the driving interactions within and between bio‐geo‐physical and human environments, as well as interactions and feedbacks between components (Ericksen, 2008; Ericksen *et al*., 2010). According to FAO (2018), food systems are intricate webs of interlinked activities and feedback loops, impacted by social, environmental and economic changes. Food systems theory and frameworks have been used to understand sudden changes or systemic shocks to food systems. Changes in food production have been investigated by Huff *et al*. (2015) using a system dynamics model, shocks to food prices by Block *et al*. (2004), sudden dietary changes by Darnton‐Hill and Cogill (2010) and dietary coping strategies by Galiano and Vera‐Hernández (2008). Shocks to food systems can vary, in terms of biophysical shocks (Béné *et al*., 2015), economic shocks (Block *et al*., 2004), and health shocks (Gillespie, 2008). The COVID‐19 pandemic was initially an acute health shock that reverberated into broader production and economic shocks and associated social and policy responses (Harris *et al*., 2020). The COVID‐19 pandemic affected multiple food system drivers simultaneously, in terms of inputs and production, trade and marketing, prices and affordability, and consumer demand at local, national, and international scales (Harris *et al*., 2020).

**Figure 1:**
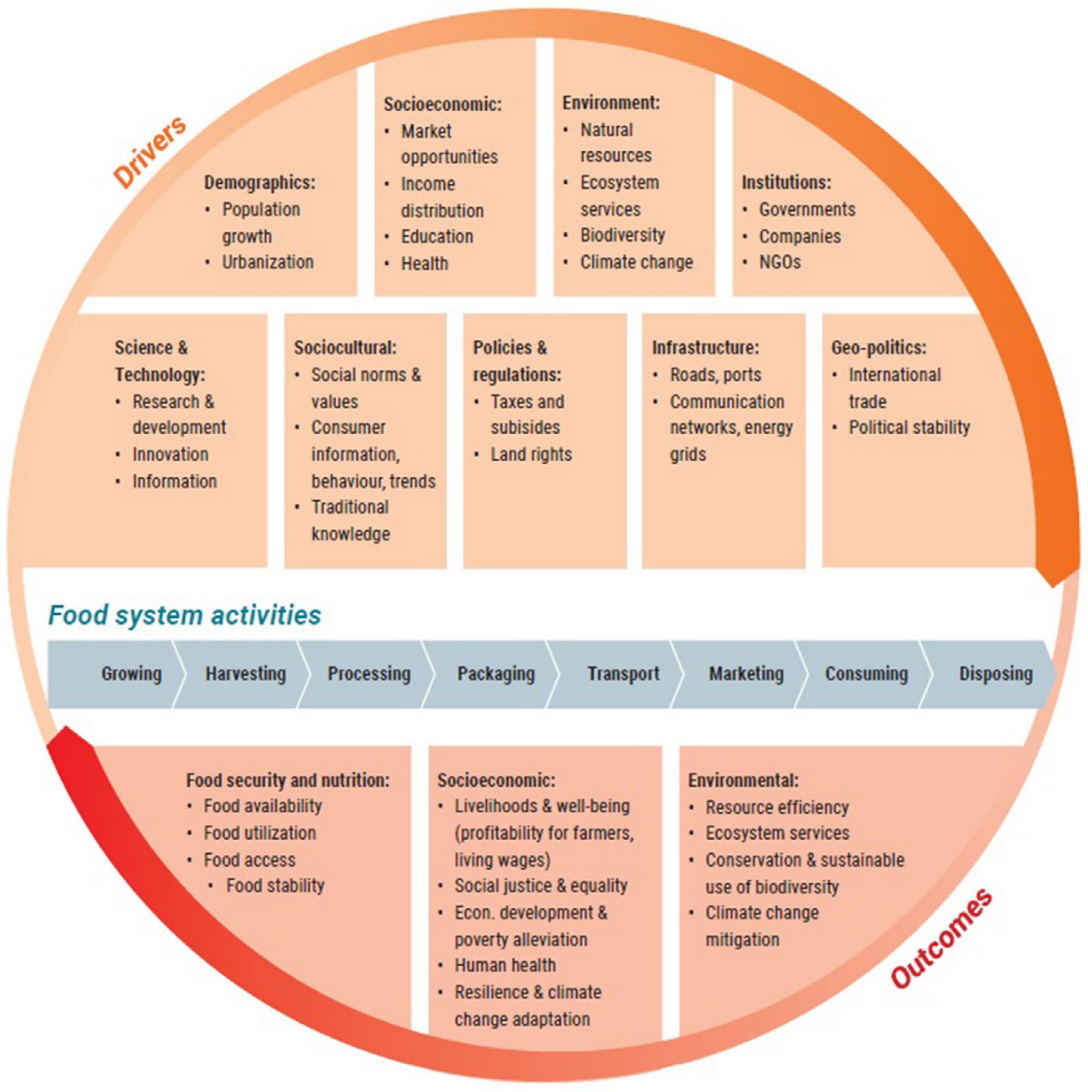
*An illustration of food systems elements, drivers, activities and outcomes*. *Source*: Bortoletti & Lomax (2019: 12).

Bortoletti and Lomax (2019) developed a food systems framework of elements, drivers, activities and outcomes as illustrated in Figure 1. Ingram (2011: 420) describes in detail the elements within food system activities that give rise to food security outcomes. In summary, a food system consists of the supply chain from farmgate to the consumer whereby outcomes influence food security, which in turn drives changes to social and environmental status. Food security and nutritional outcomes are grouped into four components (Figure 1): availability, access, utilization and food stability (Bortoletti & Lomax, 2019; Ericksen, 2008; FAO, 1996; Ingram, 2011). Food availability is related to: (1) *Production* (how much and which types of food are available through local production); (2) *Distribution* (how food is made available or physically moved, in what form, when and to whom); and (3) *Exchange* (how much of the available food is obtained through exchange mechanisms such as barter, trade, purchase or loans). Access to food is determined by: (1) *Affordability* (the purchasing power of households or communities relative to the price of food); (2) *Allocation* (the economic, social and political mechanisms governing when, where and how food can be accessed by consumers); and (3) *Preference* (social, religious or cultural norms and values that influence consumer demand for certain types of food) (Ingram, 2011). For food stability to be met, a household/individual needs to have access to an adequate and constant supply of food, income and economic resources during a year and in the longer‐term (Jägerskog & Jønch Clausen, 2012). Food utilization is determined by: (1) *Nutritional value* (how much of the daily requirements of calories, vitamins, protein and micronutrients are provided by the food people consume); (2) *Social value* (the social, religious and cultural functions and benefits food provides); and (3) *Food safety* (toxic contamination introduced during producing, processing and packaging, distribution, or marketing food; and food‐borne diseases such as salmonella and CJD) (Ingram, 2011). The COVID‐19 pandemic is notably a disrupting driver to be included in Figure 1. As such, we are particularly interested in how COVID‐19 affects food system outcomes in terms of food security and nutrition. Socio‐economic impacts are also important in maintaining food stability and influencing food security (Jägerskog & Jønch Clausen, 2012).

## Vegetable food systems

Lao PDR is a largely agrarian society and has borne significant economic and social challenges during the COVID‐19 pandemic, involving income, job and fiscal revenue losses (United Nations, 2021). Over 72 per cent of the labour force in Lao PDR is engaged in agriculture, the largest employment sector in the country (World Food Programme, 2020: 10). Lao PDR is experiencing agrarian transitional changes that are occurring elsewhere in Southeast Asia with diversification of smallholder farming activities, opportunities for contract farming, emergence of agribusiness and opportunities to secure off‐farm income (Drahmoune, 2013). Moglia *et al*. (2020) claim that modernization, commercialization and diversification of livelihoods are driving social and economic changes to traditional lifestyles.

Vegetables are an important element in diets. Vegetable production occurs largely at the household level providing diverse food and nutrients that can be sold as cash income. In Lao PDR, interest in vegetable production has recently increased. For example, in 2019, vegetable production increased to 160 000 hectares, with increasing contract farming instigated by foreign companies that provide farmers with seeds, fertilizers, plastic sheeting and irrigation (FAO, 2020). Alexander *et al*. (2017) described in detail the variety of contracts that have been accessed by smallholder farmers in Xiengkhoung Province. Contract farming can include provision of inputs, farmer group organization, provision of additional labour, technical instruction, access to credit and an assured purchase guarantee. Vegetable production is largely overseen by women, augmenting household incomes, and providing dietary micronutrients.

Dixon *et al*. (2021) found that while fruit and vegetables remained available in local rural markets in Asia during the COVID‐19 pandemic, larger market chains were significantly disrupted in many agri‐food systems. They found that traditional market chains were more resilient during the COVID‐19 lockdown due to lower population densities, lower input requirements and lower productivity. Yet Dixon *et al*. (2021) claim there is a need for recovery policies and precautionary strategies to protect against future pandemics in Asia and globally. Studies by Middendorf *et al*. (2021) in Africa found that key impacts of COVID‐19 on vegetable systems were: reduced access to inputs and to local and urban markets, reduced transportation, lower yields, income losses and increased post‐harvest losses. Supply systems were disturbed, decreasing food security. Harris *et al*. (2020) found disruptions to vegetable food systems in India had a major impact on demand, prices and incomes. Labour shortages and marketing constraints for storage and transport also occurred (Harris *et al*., 2020).

## Research details

**Figure 2:**
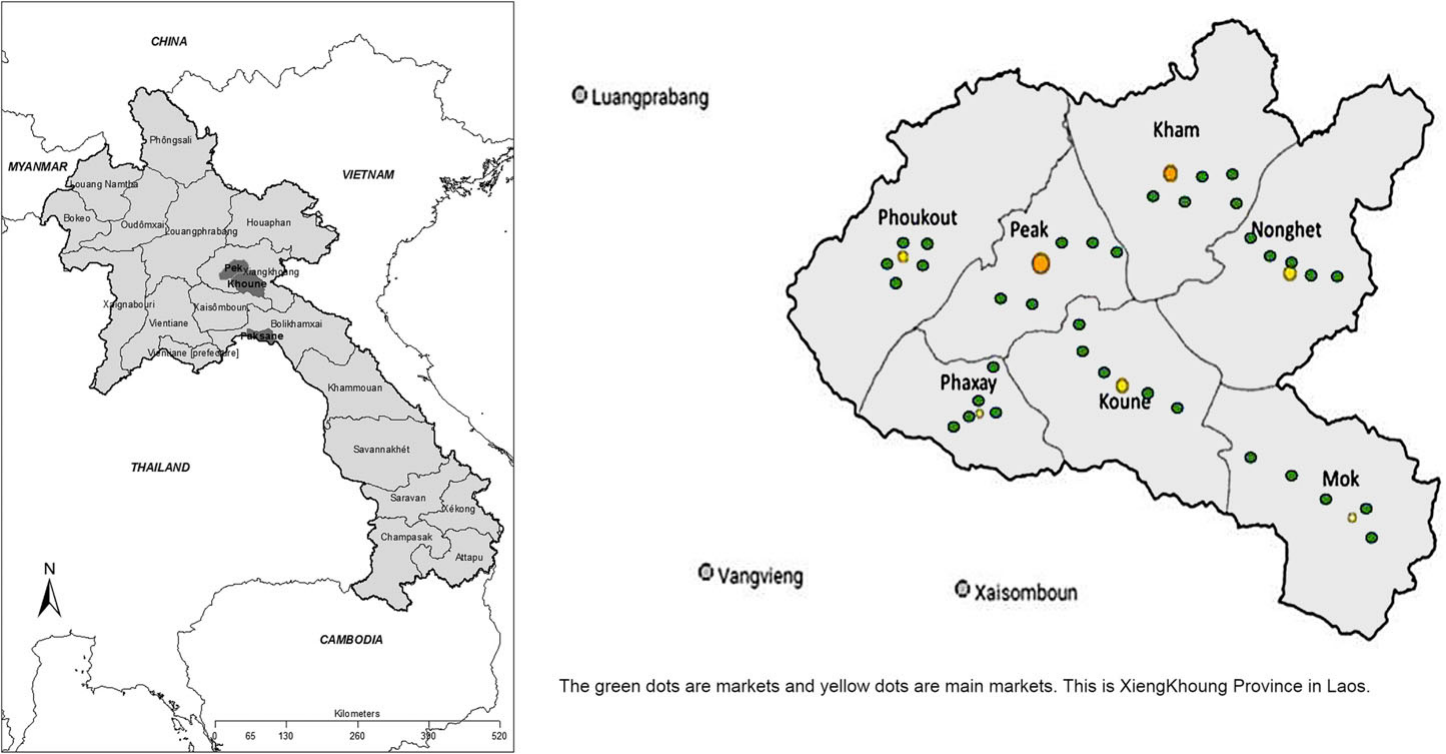
*Map of Lao PDR with location of key districts and surveyed villages in Xiengkhoung Province*. *Source*: Maps produced by Michelle Esparon (James Cook University, Townsville, Australia) and authors based on Xiengkhoung Province's population statistics, charts, map and location information available at: https://www.citypopulation.de/en/laos/admin/09__xiengkhuang/.

Research activities were situated in Xiengkhoung Province, a central province in Lao PDR contributing significantly to national agricultural production (MAF, 2015a, 2015b), and where the COVID‐19 pandemic caused significant economic and social impacts arising out of lockdowns and travelling restrictions. Xiengkhoung Province consists of seven districts, including Pek (capital), Kham, Nonghet, Khoun, Mok, Phoukoud and Phaxay districts (Figure 2). These seven districts have 40 village groups and 477 villages in total. In 2018, the population was estimated at 262 815 people with 128 888 females and 46 902 families (Lao‐Viet Cooperation Project, 2018; Rural Development and Poverty Eradication Office 2018). Approximately 70 per cent of the population live in urban areas with a population intensity of 17 people/km^2^ (Lao‐Viet Cooperation Project, 2018). Out of the regular working population, 87 per cent are engaged in agriculture and this sector contributed 37 per cent to the GDP in 2017 (Department of Labour and Social Welfare, Xiengkhoung Province, 2017). The average income for 2017 was USD 1600/person/year (Xiengkhoung Province Government, 2017). Rice farming and subsistence livelihoods prevail, supplemented with livestock rearing and the collection of non‐timber forest products (NTFPs) as well as fish from local water supplies (World Food Programme, 2020).

Vegetable production occurs largely at the household level, providing diverse food and nutrients for households and is an important means to augment household incomes (Lao‐Viet Cooperation Project, 2018). Sales are conducted directly at market or via wholesalers or retailers to local and provincial markets (Department of Labour and Social Welfare, Xiengkhoung Province 2017). Growing and selling vegetables is largely a female task, though men are involved in different processes within vegetable supply chains. Hence, research activities were largely conducted with women, and when men were interviewed, they often deferred to their wives when answering questions.

The research approach involved conducting face to face surveys with key participants in local vegetable supply chains in seven districts in Xiengkhoung Province including smallholder farmers, wholesalers, retailers and consumers. Participants were selected using a purposive technique based on information about actors, trade and markets within the food supply from the Provincial Agricultural and Forestry Officers (PAFO) in Xiengkhoung Province. The Provincial Office collates district agricultural information to inform the District Governor and national government. Official documents provided an appropriate source of information to inform purposive sampling, used to produce a representative, non‐random cross‐section sample of the population (Babbie, 2010).

Based on the selection criterion which required participants to be involved in the supply of vegetables via local markets/trading and/or agri‐businesses, 350 vegetable farmers, 60 wholesalers, 50 retailers and 70 consumers in Xiengkhoung Province were recruited. While vegetables were also transported to other districts including Vientiane Capital, Loungprabang, Oudomxay and Bolikhamxay Provinces, participants in other provincial markets were not involved in the research.

With regards to vegetable wholesalers, it is worth noting that participants were chosen from only six of the seven districts in Xiengkhoung Province as Nonghet district did not have a wholesaler, had limited vegetable markets and was located in a remote mountainous district. Producers (farmers) sold their vegetable directly to retailers or at the local market. In addition, three local markets were selected in Pek district. There are five markets in this district and Phonsavan, the district capital is the hub of economic development and sale of vegetables.

Survey questions were designed based on preliminary research involving secondary data and interviews with people involved in the local vegetable supplies. Survey questions focused on the positive and negative impacts from the March–May 2020 COVID‐19 pandemic lockdown by comparing the function of the vegetable food system before COVID‐19 and after lockdown in terms of key vegetables, quantities, prices and incomes generated. Farmers were asked about the quantity of vegetables they supplied, income, production waste, transport and demand for their produce, pricing, gender roles and business decisions. Wholesalers and retailers were asked about the quantities they bought and supplied to markets, barriers to transport, market demand (kg/day), market prices (kip/day) (1 USD = 10 005 Laotian kip), and gender roles in their business, methods of purchase, and issues and solutions they could offer if lockdowns occurred again. Consumers were asked questions regarding the type and number of vegetables they bought daily, when they visited the market, method of purchase, prices, gender roles and issues faced. Initially, survey forms were developed and piloted randomly amongst two farmers, one wholesaler, one retailer and two consumers from Xiengkhoung Province. Finalized surveys were then conducted with selected participants between March and May 2020.

## Results

### Demographics

Farmers who grew quantities of vegetables for markets were selected from 36 villages in seven districts in Xiengkhoung Province. In total, 350 farmers were involved: 85 per cent were female and 15 per cent were male, largely aged between 26 years old to 50 years old. The survey of vegetable wholesalers was conducted in six districts in Xiengkhoung Province with ten participants in each district coming to a total of 60 participants (77 per cent female and 23 per cent male). Women participated in wholesale activities from their early 20's and older women continued to be involved, while males were aged between 31 to 55 years. Vegetable retailers were selected from nine markets in Xiengkhoung Province, involving 50 active retailers. In Pek district, the capital, retailers were selected from three local markets. In other districts, retailers from each market were selected. As traditionally, mostly women sell vegetables, the sample involved women only: with over half of them ranging between the ages of 23 to 40 years. There were other women younger than 20 years and several over 61 years. In nine markets in Xiengkhoung Province, 70 consumers were surveyed (83 per cent female and 17 per cent male). Approximately, 50 per cent of consumers at the markets were aged between 36 to 45 years, and the sample ranged in age from 20 to 55 years.

### COVID‐19 impacts on vegetable food system activities

In this article, we utilized Bortoletti and Lomax's (2019) food systems framework (as described in Figure 1) as a basis for presenting our results. In Figure 1, the components of food system activities (supply chains) are: (i) growing, (ii) harvesting, (iii) processing, (iv) packaging, (v) transport (distributing), (vi) marketing (retailing), (vii) consuming, and (viii) disposing of food. Figure 1 also illustrates the food system outcomes, in terms of food security and nutritional outcomes by evaluating food availability, food access, food utilization, and food stability. As a key driver, the COVID‐19 pandemic has caused disruptions to food system activities with various outcomes in many countries. As such, we investigated vegetable food systems activities and outcomes in the study area to assess the degree and timeframe of disturbance caused to vegetable supplies by the COVID‐19 pandemic.

#### Vegetable production: Growing, harvesting, processing, packaging, transport, marketing, consuming, disposal.

A list of 26 vegetables grown and sold in Xiengkhoung Province was shown to participants. All participants indicated that the top five vegetables impacted by the COVID‐19 pandemic were: spring onion [Lao 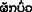] (*Allium fistulosum*); Chinese flowering cabbage or Choy sum [Lao 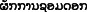] (*Brassica rapa* var. *parachinensis*); coriander [Lao 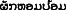] (*Coriandrum sativum)*; Chinese cabbage [Lao 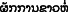] (*Brassica rapa* subsp*. Pekinensis*); and cabbage [Lao 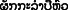] *(Brassica oleracea)*. The purpose of the survey was to determine the main impacts of the pandemic on vegetable supply chains/food systems. Notably, participants regarded the top five vegetables previously mentioned to be more likely impacted by the pandemic, as these vegetables are perishable and important to local cuisines. Consumers tended to buy these products regularly from local markets. Quantities of these vegetables were also transported by wholesalers to several other Provinces including Vientiane Capital, Loungprabang, Oudomxay and Bolikhamxay and sold into local markets.

When we compared total production quantities (kg) of the five key vegetables supplied, it was observed that while farmers (N = 350) on average produced/sold 74 175 kg/week before lockdown—during lockdown, this amount decreased to only 32 330 kg/week (56 per cent decrease) (Table 1). After lockdown, farmers supplied more vegetables, causing volumes to increase by 14 per cent (84 818 kg). Farmers from Kham and Khoun districts experienced greater volume/sale losses, as they were the main suppliers in the province (Table 1). There was a reduction in the quantity of five key vegetables supplied during lockdown to the following extents: spring onion (66 per cent), Chinese flowering cabbage (65 per cent), Chinese cabbage (56 per cent), cabbage (49 per cent), and coriander (46 per cent) (Table 1). After lockdown, all supplies increased beyond pre‐COVID levels.

**Table 1:** Quantity of vegetables supplied by farmers before, during and after lockdown (kg/day) (N = 350).

Vegetables supplied (kg/day/district)	Time period	Cabbage	Coriander	Chinese Flowering Cabbage	Chinese Cabbage	Spring onion	Total supplies (kg/day)
Pek	Pre‐COVID‐19	450	807	785	454	773	3269
	Lockdown	77	194	196	93	198	758
	Post‐COVID‐19	500	871	805	538	814	3528
Kham	Pre‐COVID‐19	4621	3100	7474	6948	8183	30326
	Lockdown	545	1764	1341	1660	2349	7659
	Post‐COVID‐19	6233	3434	9443	8915	10268	38293
Nonghet	Pre‐COVID‐19	317	612	803	412	642	2786
	Lockdown	112	275	343	199	301	1230
	Post‐COVID‐19	163	352	418	229	346	1508
Khoun	Pre‐COVID‐19	9726	1560	1400	10151	1089	23926
	Lockdown	7108	1763	808	6088	930	16697
	Post‐COVID‐19	10364	1812	1184	10564	1117	25041
Mok	Pre‐COVID‐19	1244	1519	1729	1543	1147	7182
	Lockdown	730	848	964	888	609	4039
	Post‐COVID‐19	1717	1958	2317	2107	1225	9324
Phukhut	Pre‐COVID‐19	380	883	814	742	805	3624
	Lockdown	10	64	71	87	61	293
	Post‐COVID‐19	322	722	692	580	674	2990
Phaxay	Pre‐COVID‐19	66	978	873	407	738	3062
	Lockdown	20	209	180	92	153	654
	Post‐COVID‐19	91	1323	1174	528	1018	4134

*Source*: Table produced by authors based on field research data.

#### Distributing and retailing.

Ingram (2011) describes retailing and distributing as a key component of food system activities (Figure 1). Retailing and distributing usually includes a range of middlemen who go between the producers, processors, packers and the final markets—and the many actors involved in transport, delivery and warehousing operations, advertising, trading and supermarkets (Ingram, 2011: 420). In this research study, we surveyed wholesalers, retailers and farmers who sold produce directly at market.

#### Wholesale activities

Vegetables produced on the farm were often sold to wholesalers, though sometimes produce was sold at markets by female farmers. Changes to wholesale vegetable trading (average kg/day) were based on average trading activities of the five key vegetables (cabbage, coriander, Chinese flowering cabbage, Chinese cabbage, and spring onion) in seven districts. During lockdown, the total average trading (kg/day) reduced by 54 per cent and was still below normal levels (by 2 per cent) after the COVID‐19 lockdown. In local markets, usually 38 999 kg/day of the five vegetables were traded pre lockdown, but during lockdown, supplies reduced to 19 694 kg/day (‐51 per cent), returning to 38 343 kg/day after lockdown. In provincial markets, supplies of the five vegetables traded were usually 102 215 kg/day pre lockdown, but during lockdown, supplies reduced to 45 917 kg/day (‐55 per cent). As the wholesalers market to other provincial markets, the COVID‐19 pandemic clearly affected local and provincial vegetable markets. Significant falls in vegetable supplies suggest that food security and nutrition was challenged.

Wholesalers traded or ‘exchanged’ vegetables with variously‐sized farmer groups. Before lockdown, half the farmer groups involved 10–30 farmers, with 38 per cent of groups having less than 10 farmers and 12 per cent having up to 50 farmers. During lockdown, wholesalers more often bought produce from groups of less than 10 farmers (67 per cent) and from groups of 10–30 farmers (32 per cent). Further, it was observed that during lockdown, wholesalers contacted farmer groups to sell or order vegetables by phone (85 per cent), face‐to‐face contact was reduced from 27 per cent to 8 per cent, and online contact increased from 2 per cent to 7 per cent.

#### Retail activities

Local female retailers sold vegetables in the fresh markets in Xiengkhoung Province, where the tasks involved: ordering, picking up, displaying and selling vegetables. Husbands rarely assisted in retail activities. Women tended to set selling prices for vegetables more so than men before COVID‐19 lockdowns, though during and after lockdown, more husbands and wives made decisions on prices together. Sale and purchase of vegetables by retailers during lockdown more than halved (9286 kg/day reduced to 3903 kg/day). Thereafter, markets stabilized to pre‐lockdown levels. Clearly, during lockdown, retail sales of the main vegetables produced and exchanged were significantly and negatively impacted. Retailers selling at the local market faced similar problems as did wholesalers, due to supply chain contractions. Disruption to trade affected incomes for many people and businesses involved in the supply of vegetables.

Whereas before lockdown, retailers traded or ‘exchanged’ vegetables with five or fewer wholesalers (50 per cent), during lockdown, they made purchases almost exclusively with five or fewer wholesalers (90 per cent). This appeared to be a risk mitigation strategy. Retailers also usually contacted wholesalers to order vegetables by phone (54 per cent) before lockdown; however during lockdown, phone contact increased to 82 per cent of orders with online contact increasing from 6 per cent to 16 per cent.

#### Consumption

Consumers were usually women buying fresh vegetables at the markets. Although vegetables are perishable, they provide important nutrients in rice‐based diets. The quantities of the five vegetables bought by consumers (kg/day) remained steady during lockdown and increased by 3.5 per cent after lockdown. Consumers (N = 70) bought the same weight of vegetables (~12kg/day) before, during and after lockdown. During lockdown, each time they visited the market, consumers bought slightly more cabbage and flowering cabbage than they would have bought before the COVID‐19 pandemic. For example, while Chinese cabbage purchases on average increased from 1.5 kg/visit before COVID‐19, during lockdown consumers bought 2.4 kg/visit. Consumers changed their purchasing habits slightly by shopping less frequently during lockdown. More of them were looking to purchase vegetables by phone and through other online sources during lockdown. Regular sales resumed after lockdown.

### COVID‐19 impacts on vegetable food system outcomes

#### Food availability—production, distribution, exchange.

The three‐month lockdown affected the availability of food products, notably in terms of production, distribution and exchange of food. All participants in vegetable food supply chains in Xiengkhoung Province claimed they faced problems during the COVID‐19 pandemic lockdown. Farmers mentioned many difficulties impacting food availability during lockdown, some of which include: difficulties in going out and selling vegetables, few buyers, reduced demand and lower prices for produce, market uncertainty, problematic logistics, income losses, lack of government support, curtailed movements, and expensive and inaccessible agricultural inputs.

Wholesalers faced similar problems to farmers (producers) during lockdown. They were predominantly concerned about: difficulties in going to the market and transporting vegetables; problems obtaining transport permission from the COVID‐19 prevention committee; difficulties in collecting vegetables from farmers; wasted produce; and their falling incomes. To mitigate these problems, wholesalers sold at lower prices; reduced or stopped purchasing vegetables from farmers; fed leftover vegetables to livestock; obtained transport permission letters from the COVID‐19 prevention committee; shared or exchanged produce with others; offered delivery services through social media; or made compost from left‐over vegetables.

Retailers faced similar problems as wholesalers and farmers. The three main problems for retailers were: difficulties in going out to buy and sell vegetables (28 per cent), fall in income (22 per cent), and fewer buyers at markets (20 per cent). Common responses to these issues included: use of leftover vegetables to feed livestock (29 per cent); selling produce at lower prices (23 per cent); refraining from purchasing produce from farmers and wholesalers (21 per cent); and sharing and exchanging vegetables with others (10 per cent). In addition, 6 per cent of retailers used social media to sell and deliver vegetables to consumers.

The extensive lockdown created many issues for consumers: they faced difficulties accessing the market to buy food and vegetables (29 per cent); household expenditure such as food, water, electricity and communication costs (internet, phone and delivery) increased (21 per cent); there was insufficient income for expenditure (20 per cent); they faced increased prices of food and vegetables (20 per cent); and were unable to store food and vegetables for long periods of time (10 per cent).

#### Access (affordability, allocation, preference).

Ericksen (2008) suggests that food access is a function of conversion of various financial, political and other assets into food, whether produced or purchased. Consequently, inequity in food distribution and allocation is based on income, political and social power. Hence, income is a primary determinant of consumption and food security status (Ericksen, 2008).

#### Incomes

Reduced prices for farmers created hardship, particularly if their incomes were totally reliant on the sale of vegetable produce. Farmers' incomes reduced significantly during lockdown. Before lockdown, 97 per cent of farmers had incomes greater than 301 000 kip/week. However, during lockdown, only 33 per cent of farmers were able to attain that level of income. Wholesalers also faced income losses. For 59 per cent of wholesalers, incomes dropped below 500 000kip/week during lockdown. Before lockdown, 43 per cent of wholesalers had incomes greater than 2 000 000 kip/week, a figure that was impossible to achieve during lockdown. Retailers also faced a similar situation: before lockdown, only 6 per cent of retailers relied on an income of less than 300 000 kip/week, yet during lockdown, 66 per cent of retailers had to cope with this lower income. The maximum income for retailers during lockdown was 800 000 kip/week, significantly lesser than usual incomes of up to 2 000 000 kip/week prior to lockdowns.

#### Vegetable prices

The price of vegetables is an indication of food affordability. Prices of vegetables are seasonal and influenced by demand and supply. Price volatility often depends on the food. Uncertainties during the COVID‐19 pandemic in turn influences volatility. During the COVID‐19 lockdown, demand for vegetables declined, impacting prices throughout the vegetable supply chain. Farmers continued to produce vegetables for the market although they were only able to sell half of the usual amount of produce to wholesalers. Prior to COVID‐19, 8 per cent of wholesalers bought vegetables from farmers in the lower price range of 200–1000 kip/kg; this increased to 22 per cent of wholesalers during lockdown. Fewer wholesalers bought vegetables in the higher price ranges during lockdown. Wholesalers were also unable to on‐sell the vegetables and/or had to sell at lower prices as compared to before the COVID‐19 pandemic.

In the local fresh markets within the province, the number of retailers who generally bought at lower prices (1000–3500 kip/kg) doubled from 21 per cent before COVID‐19 to 45 per cent during lockdown. Retailers had fewer higher priced sales. Approximately 74 per cent of retailers said they sold at lower prices during lockdown than before lockdown. Hence, retailers experienced a similar situation with wholesalers—low demand resulted in fewer purchases and at lower prices, with some retailers withdrawing from the market. After lockdown, buying habits reflected pre‐COVID‐19 price ranges.

When asked, 20 per cent of consumers claimed that generally, the prices of food and vegetables increased during lockdown. Before lockdown, on average, 50 per cent of vegetables were sold for up to 5000 kip/kg with 47 per cent sold for 10 000 kip/kg. During lockdown, consumers on average bought fewer vegetables priced at 10 000 kip and more vegetables priced above 15 000 kip as compared to before or after COVID‐19 lockdown. For example, 2 per cent of consumers bought cabbage at 25 000 kip/kg or above before the COVID‐19 pandemic, but during lockdown, 13 per cent of consumers bought cabbage in this price range. Prices stabilized after the COVID‐19 lockdown.

All stakeholders along the vegetable supply chain in Xiengkhoung Province were affected by changes to vegetable quantities and prices. Wholesalers and retailers managed sales, traded out of difficulties or ceased trading, while farmers sold less of their produce at lower prices and consumers paid slightly higher prices for some vegetables.

#### Environmental impacts.

Local ecosystems provide dietary supplements (NTFPs) if populations experience food insecurity or need to supplement diets. Hence, local ecosystems are a safety net when food insecurity occurs. Environmental impacts could arise from wasted use of ecosystem services (land, water, soil etc) in terms of food wastage. Changes to environmental impacts did not feature in our research as the focus was on food activities and outcomes impacted by COVID‐19 lockdowns.

#### Socio‐economic impacts.

Most respondents indicated that the key positive benefits of the lockdown were: changes in household and social expenditure as social leisure activities and movement were curtailed; an opportunity to spend more time with their family; more time to tend to their gardens; and developing more of an interest in establishing online selling/buying opportunities.

An important element of social welfare is social capital, i.e. the relationships among people who live and work in a particular society, enabling that society to function effectively. Throughout the COVID‐19 lockdown, women continued to make decisions about vegetable prices for the market, although they did confer with their husbands, particularly during the lockdown. Changes to the time and labour spent producing vegetables also occurred. At the farmgate, production of vegetables included a variety of tasks and on average, women and men worked longer in their gardens during and after the COVID‐19 lockdown.

Women were largely involved in wholesaling activities, processing orders, collecting and packing vegetables, and delivery of vegetables. Husbands and wives tended to work together taking orders more so during and after lockdown. Retailers, of which 96 per cent were female, processed orders and collected, displayed and sold vegetables. Notably, although the COVID‐19 lockdown reduced retail opportunities, women continued to manage their businesses.

Table 2 indicates the overall socio‐economic effects of the COVID‐19 lockdown on the supply of the five key vegetables. The lockdown had a major, though short‐term impact on all participants in the vegetable supply chain, impacting their socio‐economic status. While smallholder vegetable growers were vulnerable to supply chain fluctuations due to the perishability of their products, the COVID‐19 lockdown was a disruptive driver (Figure 1).

**Table 2:** Summary of food activities and outcomes.

Food system participant and *food activity*	Purchases Average kg/day	Sales Average kg/day	Prices Average kip/kg	Incomes Average kip	Overall Outcome
Farmers *Producers*	Inputs more expensive	Sales to wholesalers and direct to local market (‐56%)	Prices reduced (‐18%)	Reduced income (‐70%)	Reduced demand Lower prices Reduced incomes
Wholesalers *Packaging* *Transport* *Marketing*	Purchases from farmers reduced (‐50%)	Sales reduced at provincial markets (‐55%) and local markets (‐50%)	All prices reduced	Reduced incomes (‐43%)	Reduced purchases Reduced demand Fewer sales
Retailers *Packaging* *Transport* *Marketing*	Purchases from wholesalers reduced (‐58%)	Sales reduced at local markets (‐58%)	Prices dropped (‐17%)	Reduced incomes (‐86%)	Reduced purchases Reduced demand Fewer sales
Consumers *Consumption*	Little change to purchases	Would like to grow their own vegetable supplies in the case of future lockdowns	Reduced supply and increase in prices	Concerned about their income during lockdown	Limited access to markets Purchasing habits changed Reduced supply Some higher prices

*Source:* Table produced by authors based on field research data.

#### Another lockdown—coping, mitigation, and resilience.

Should another lockdown occur, farmers expressed major concerns including loss of income, market uncertainty, fewer buyers for their produce, and having to lower sale prices. Farmers offered several solutions should lockdown occur again and they included: changing production decisions and moving into integrated farming, where a more diverse array of produce can be farmed; improving garden planting plans; contacting wholesalers directly; and creating an online selling platform.

The concerns that wholesalers expressed included: increased vegetable prices, lower incomes, and difficulties in transporting vegetables to other provinces. Several options to address these concerns included: planning and contracting with farmers; improving packaging for transport; and creating online selling.

Retailers were concerned about market uncertainty, fewer buyers at markets, low‐priced sales, and health concerns about contracting COVID‐19. To deal with these concerns, they mentioned: increasing personal safety; obtaining permissions to continue selling at the market; and creating an online selling platform.

Consumers claimed that with another lockdown, they may need to create a household garden to provide and store fresh vegetables, if possible, for their families. They were also concerned about managing their incomes and increasing their savings.

## Discussion

### Impacts on vegetable food systems in Xiengkhoung Province, Lao PDR

In a largely agrarian society like Laos, rural livelihoods are subsistence‐based and supplemented by income‐based activities where possible. Traditional farming systems feature small‐scale production, processing, and trade, and market infrastructure is rudimentary (Stefanovic *et al*., 2020). Rural households in traditional farming systems rely on their own seasonal food production, and food stability is more important than yield maximization (Ericksen, 2008). Dixon *et al*. (2021) claim that traditional farming systems in Asia Pacific were found to be more resilient during the COVID‐19 lockdown due to lower population densities, lower input requirements and lower productivity. In Xiengkhoung Province, while incomes drastically declined for actors in the food system, livelihoods resumed as soon as the lockdown was lifted.

Trade of five key vegetables in Xiengkhoung Province was impacted by the COVID‐19 pandemic constraints. Overall findings are as follows. Farmers (producers) found inputs more expensive, and experienced reductions in demand, sales, prices and incomes. Produce sales reduced by 56 per cent, prices reduced by 18 per cent, and incomes reduced by 70 per cent. Reduced prices for farmers created hardship, particularly when their incomes were totally reliant on sale of vegetable produce. Disruptions occurred in packaging, transport and marketing processes of food activities with impacts on wholesalers and retailers involved in the supply chain. Wholesalers reduced their purchases from farmers by 50 per cent and market sales declined (at local markets by 50 per cent and at provincial markets by 55 per cent). Wholesalers reduced prices and their incomes decreased by 43 per cent. Retailers reduced their vegetable purchases from wholesalers by 58 per cent; similarly sales at local markets reduced by 58 per cent. Retailers reduced prices by 17 per cent and their incomes decreased by 86 per cent. Less demand and fewer sales significantly impacted retailers. Consumers experienced a reduced supply of vegetables and had to purchase some vegetables at higher prices. They also had more limited access to markets that led to a change in purchasing habits. All actors in the food system reported that production, sales, prices, availability and incomes returned to pre‐COVID‐19 levels after lockdown.

The COVID‐19 pandemic initiated an additional food system driver or ‘shock’ to this traditional farming/food system. Vegetables are an important part of the dietary cuisine, and a supply of fresh vegetables is very important in terms of nutritional components, dietary preferences and as vital ingredients to traditional recipes. According to Harris *et al*. (2020), a focus on vegetable production provides a snapshot of the disruption impacts on a source of nutritious foods. In our research, we found the impact on vegetable food systems revealed a general collapse in each area of the supply chain and then reinstatement of the traditional food system trading activities, without long‐term implications. While the lockdown was imposed over a three‐month period, vegetables were produced, and food was available and accessible in local and provincial markets. Vegetable demand diminished temporarily, and unsold produce was utilized in other ways. Food stability was impacted as the COVID‐19 disruptions interrupted food supplies. However, food availability and access resumed post‐lockdown, indicating only temporary impacts. While producers, wholesalers and retailers experienced a significant and temporary drop in income, their livelihoods resumed post‐lockdown.

As we used a case study approach, our findings may not be generalizable to other farmers, crops, markets or households relying on other livelihoods. Yet the literature indicates similar impacts occurred in other areas of the traditional food system chain level (Market Development Facility, 2021; OECD, 2020a; Sidaway, 2021). The lockdown had a major, though short‐term impact on all participants in the vegetable supply chain, disrupting their socio‐economic status, consumption patterns and food security status.

The price of vegetables is an indication of food affordability. Vegetable prices vary due to seasonal production and supply and demand. Vegetable prices are also often complicated by production/sales uncertainties as experienced in the COVID‐19 pandemic lockdown where demand for vegetables reduced, impacting prices throughout the vegetable supply chain. Farmers continued to produce vegetables for the market although they were only able to sell half of the usual amount of produce to wholesalers. Produce was available, but trading issues disrupted the supply chain.

Women as producers, wholesalers, retailers and consumers found their businesses more affected by the lockdown. They continued to make decisions about vegetable prices. Husbands tended to support the business enterprise, over their normal roles in terms of effort, decisions and involvement. While women experienced income losses, this was temporary and nutritional status was maintained through traditional subsistence strategies of food sharing, feeding livestock and delaying harvest.

Participants in the vegetable food system described actions they would take to mitigate problems if another lockdown occurred. These actions represent socio‐economic risk mitigation strategies. Suggestions involved planning and contracting sales, modifying transport, packaging and production, changing methods of contact, authorizing permission, reigning in spending habits, and creating an online selling platform.

### COVID‐19 pandemic food system disruptions

Almost all countries have experienced disruptions from COVID‐19 interventions affecting food production and demand, and adversely affecting food availability (OECD, 2020a; *UN News*, 2020). Health, business and agricultural sectors continue to be significantly affected (CIDRAP, 2022). Reports on vegetable production by Alam and Khatun (2021), Harris *et al*. (2020) and Middendorf *et al*. (2021) indicated the key impacts depended on the scale of production (farm size), the diversity of vegetables, access to markets and access and transportation for goods. Negative impacts affected production, sales, prices and incomes, which in turn increased vulnerability and food insecurity.

As Lao PDR is in the grip of agrarian transitional changes (Drahmoune, 2013) encompassing production diversification, contract farming, agribusiness and opportunities for off‐farm income, the COVID‐19 pandemic has temporarily paused these processes and added to the burden of risk for smallholder farmers. As found by Harris *et al*. (2020), smallholder farmers in Lao PDR resorted to social strategies to deal with changes to demand as they consumed their own produce, did not harvest, fed vegetables to livestock, and shared vegetables with other families. However, smaller farms were less likely to experience major disruptions, reflecting the traditional production tactics, using social safety nets, and balancing food stability through local consumption. Hence, food insecurity was averted as the duration of the disruption was only three months. Farming households withstood the disruption by accepting lower prices to maintain sales in the short term and minimize food shortages.

Alam and Khatun (2021) suggest that cash support is a vital strategy, and provision of low‐cost resources are required to mitigate food shortages resulting from pandemics. Our synthesis supports Dixon *et al*. (2021), in suggesting that traditional food systems and market chains can be resilient, when there are lower population densities, lower input requirements and lower productivity. This, however, does not negate the need to consider recovery policies and precautionary strategies to aid smallholder farmers should the crisis amplify. In Lao PDR, IWMI & NAFRI (2021) suggest providing seed kits, home gardening equipment, animal healthcare material and technical support through District Agriculture and Forestry Officers and local service providers to prepare households for future lockdowns. ADB (2020) suggests protecting communities affected by COVID‐19's health and economic impacts by enhancing food security and invoking support services, such as social protection programs.

As all participants in the food system in Xiengkhoung Province suggested they would use online mitigation strategies, development of digital platforms and use of agricultural technology would support these strategies. In addition, ADB (2020) claims reforms are required to strengthen supply‐chain management, particularly for labour‐intensive supply chains. It may be pertinent to consider the need to overcome rural challenges, through agricultural, institutional and legislative and policy reforms to deal with future disruptions to supply chains (ADB, 2020; Barbier & Burgess, 2020; Gupte & Mitlin, 2021). Maintaining the agri‐food systems is a priority according to Zhan and Chen (2021) who suggest it is important to enact targeted policies supporting businesses and households.

In Lao PDR, IWMI and NAFRI (2021) suggest that the government's agricultural policy responses to COVID‐19 requires a review of the nation's socioeconomic development plan. Many rural populations are living in poverty and are often food insecure in certain times during the year. Policies are required to lift these populations out of poverty (World Food Programme, 2020). Of particular concern is the reliance on natural resources in rural areas during times of hardship. More importantly, efforts to assist smallholder farmers in dealing with threats such as COVID‐19 are needed to protect these important and sustaining resources from being over‐harvested.

## Conclusion

The COVID‐19 pandemic lockdown in 2020 significantly disrupted vegetable food systems in Xiengkhoung Province, Lao PDR. From a food system perspective (Figure 1), the COVID‐19 pandemic lockdown drove changes to markets, income and health (socio‐economic impacts); involved government institutions, policies and trade relations (geo‐political); increased infrastructure requirements; changed sociocultural behaviours; and relied on technology to provide solutions. These drivers impacted business‐as‐usual vegetable food system activities (supply chain) with various temporary socioeconomic, environmental and food security outcomes for rural and urban populations. Yet food security was maintained through the lockdowns as, while the impact was immediate, the timeframe of the disturbance to vegetable supplies was minimal and food system activities resumed.

In Lao PDR, vegetable food systems incorporate small‐scale production, processing and trade, within rudimentary market infrastructure. Actors involved in the production and sale of vegetables immediately experienced impacts to their ability to trade and consequently faced reductions in incomes, prices, purchases, transport and sales of produce. However, livelihoods resumed as soon as the lockdown was lifted. Traditionally, vegetable production and trading are a woman's tasks and hence women were the most affected by the disruptions. As trade drastically contracted during the COVID‐19 lockdown, the stability of the food supply was threatened, but only temporarily, indicating that a traditional, resilient farming system based on lower population densities, lower input requirements and lower productivity could adapt to disruptions. To maintain resilience, supportive services and policies could be considered. Development of online communications would be useful to ensure that food supply chains remain viable should future disruptions occur.
